# Oxidative stress, depressive symptoms, disability, cognitive outcomes and hand dexterity in relapsing-remitting multiple sclerosis patients with mild/moderate disability

**DOI:** 10.1016/j.ibneur.2026.07.018

**Published:** 2026-07-31

**Authors:** Reza Mosaddeghi-Heris, Niloofar Taheri, Mahnaz Talebi, Amirreza Naseri

**Affiliations:** a Research Center for Integrative Medicine in Aging, Aging Research Institute, Tabriz University of Medical Sciences, Tabriz, Iran; b Department of Psychiatry and Behavioral Sciences, Stanford University, Palo Alto, CA, USA; c Neurosciences Research Center (NSRC), Tabriz University of Medical Sciences, Tabriz, Iran; d Student Research Committee, Tabriz University of Medical Sciences, Tabriz, Iran; e Research Center for Evidence-Based Medicine, Iranian EBM Centre: A Joanna Briggs Institute (JBI) Center of Excellence, Tabriz University of Medical Sciences, Tabriz, Iran; f Tabriz USERN Office, Universal Scientific Education and Research Network (USERN), Tabriz, Iran

**Keywords:** Cognitive impairment, Multiple sclerosis, Disability, Oxidative stress, Depression, Processing speed, Working memory, Neuropsychology

## Abstract

**Background:**

This study aimed to comprehensively examine the interplay between demographic factors, peripheral oxidative stress markers, disability, depressive symptoms, cognitive outcomes, and hand dexterity in Relapsing-remitting Multiple sclerosis (RRMS) patients with mild/moderate disability.

**Methods:**

In this cross-sectional study, RRMS patients with Expanded Disability Status Scale (EDSS)≤ 3 underwent assessments of cognitive function (Symbol Digit Modalities Test [SDMT], Paced Auditory Serial Addition Test [PASAT-3]), depressive symptoms (Beck Depression Inventory-II [BDI-II]), hand dexterity (Nine-Hole Peg Test [9HPT]), and neurological disability (EDSS). Oxidative stress markers (malondialdehyde [MDA], total antioxidant capacity [TAC], superoxide dismutase [SOD], catalase activity [CAT], glutathione peroxidase [GPx], and reduced glutathione [GSH]) were also measured.

**Results:**

The studied cohort comprised 83 patients (77.1% females; mean age: 32.65 ± 8.05 years). In addition to negative association between the EDSS and cognitive outcomes in binary logistic regression analysis (SDMT: OR=0.95; p = 0.02; PASAT: OR=0.92; p = 0.01); neurological disability also correlated with cognitive scores in multivariable linear regression (SDMT: β=−0.23, p = 0.047; PASAT: β=−0.36, p = 0.002). Education also correlated with the cognitive outcomes (SDMT: β=0.29, p < 0.001; PASAT: β=0.27, p = 0.01). MDA was associated with abnormal cognitive function (SDMT≤44; OR: 2.98 (95%CI: 1.33, 6.66) but correlation between these outcomes was no longer significant in multivariable linear regression (β=-0.21; p = 0.051). Except for a marginal correlation between EDSS and MDA (β=0.21, p = 0.04) and between 9HPT-M and TAC (β=0.26, p = 0.01), there was no significant finding regarding the correlations between the clinical and laboratory outcomes.

**Conclusion:**

Even in patients with mild/moderate disability, EDSS and education level were emerged as the strongest predictors of cognitive outcomes in RRMS.

## Introduction

Multiple sclerosis (MS) is a chronic immune-mediated disease of the central nervous system (CNS) characterized by inflammation, demyelination, and progressive axonal loss (Boutitah-Benyaich et al., 2025). Relapsing-remitting MS (RRMS) is the most common phenotype. While historically conceptualized as a disease of motor and sensory disability, cognitive impairment now ranks among the most disabling and employment-limiting features of MS, affecting up to two-thirds of patients across disease stages (Benedict et al., 2020). Deficits in information processing speed and working memory represent the cognitive signature of MS, often preceding detectable changes in physical disability and substantially impairing occupational functioning and quality of life (Askari et al., 2024, Benedict et al., 2020, Talebi et al., 2022, Wu et al., 2025). Depressive symptoms are also common in MS patients and suggested as the most common psychological comorbidity (Sullivan et al., 2025).

Oxidative stress, defined as an imbalance between pro-oxidant generation (reactive oxygen species (ROS) and reactive nitrogen species (RNS)) and antioxidant capacity, has been mechanistically implicated in MS pathogenesis (Jiménez-Jiménez et al., 2024, Jomova et al., 2023). In details, malondialdehyde (MDA) causes cytotoxicity through mechanisms involving protein cross-linking, DNA binding, and mitochondrial dysfunction. Mitochondrial glutathione protects against oxidative stress by detoxifying ROS and preserving mitochondrial function; therefore, glutathione dysregulation impairs energy metabolism and may also disrupt T-cell function by altering ROS-dependent immune signaling. Superoxide dismutase (SOD), catalase (CAT), and glutathione peroxidase (GPX) are also key antioxidant enzymes that protect against oxidative stress by converting reactive oxygen species into less harmful molecules (Alipour et al., 2026, Bizoń et al., 2023, Zhang et al., 2020).

Evidence have motivated the hypothesis that systemic oxidative imbalance may serve as a contributing factor in MS progression and consequences (Yuan et al., 2026). A recent systematic review reported inconsistency regarding the possible association between the TAC, and MDA and MS-related cognitive outcomes, based on limited available evidence. Also, GPX, reduced glutathione (GSH), and CAT was not found to be associated with cognitive findings in MS (Daneshvar et al., 2025, Naseri et al., 2022, Talebi et al., 2021). Recently, in a cross-sectional study of 77 RRMS patients, the correlation between markers for oxidative stress and the presence of associated symptoms, such as depression and cognitive impairment was evaluated. SOD activity, TAC, and glutathion reductase activity were assessed as markers of the antioxidant capacity and 4-Hydroxynonenal (4-HNE) and 8-hydroxy 2 deoxyguanosine (8-OHdG) were measured to as markers of oxidative stress. Multiple linear regression analysis revealed marginal positive linear correlation between Expanded Disability Status Scale (EDSS) and glutathion reductase; however, no significant correlation was found between EDSS and SOD, TAC, 4-HNE, and 8-OHdG. Median EDSS in this study was 2 (<2 in 49.4%, 2–5 in 44.2%, and >5 in 6.5%). In addition, no significant association was reported regarding the depressive symptoms. Impaired information process speed (defined as Symbol Digit Modalities Test [SDMT]≤38) was found to correlate with age, level of education, EDSS, and Nine-Hole Peg Test (9HPT). Regarding the biochemical outcomes, correlation between 4-HNE and TAC with processing speed were also reported (Piñar-Morales et al., 2025).

Despite advances in the understanding of MS-related physical and neuropsychological consequences, a critical gap exists in the MS literature regarding the relative importance of different pathobiological and demographic factors. The present study was designed to comprehensively investigate the relationships between demographic factors, peripheral oxidative stress markers, disability, depressive symptoms, cognitive outcomes, and hand dexterity in a cohort of patients with RRMS with mild/moderate disability (EDSS≤3).

## Methods

### Study Design and Participants

This cross-sectional study enrolled 83 consecutive patients with RRMS diagnosed according to the 2017 McDonald criteria (Thompson et al., 2018), recruited from a tertiary MS specialty clinic in Tabriz, Iran. Inclusion criteria were: (1) definite RRMS diagnosis, (2) age ≥ 18 years, (3) clinical stability defined as no clinical relapse or corticosteroid treatment within four months prior to enrollment, (4) literacy level of ≥ 9 years, (5) mild/moderate disability (EDSS≤3), and (6) informed consent. Exclusion criteria included: (1) other concurrent neurological, systemic inflammatory, diagnosed psychiatric, or metabolic disorders, (2) active infection within four months prior to enrollment, and (3) history of alcohol abuse or addiction. The protocol for this study was approved by the ethics committee of Tabriz University of Medical Sciences.

### Clinical and Neuropsychological Assessments

Level of disability was quantified using the Kurtzke's EDSS (Kurtzke, 1983), by an experienced neurologist. Other assessments were performed by trained personnels under standardized conditions on a single visit day. Cognitive performance was assessed using two complementary validated instruments sensitive to MS-related cognitive dysfunction: SDMT (Benedict et al., 2017) with ≤ 44 as a cut-off for abnormal cognitive function (Beier et al., 2017), measures visual information processing speed and requires sustained attention (Nasiri et al., 2023) and Paced Auditory Serial Addition Test (PASAT-3s) (Wiens et al., 1997), assesses working memory and auditory information processing speed (Naseri, Forghani, et al., 2022). Manual dexterity was assessed with the 9HPT (Solaro et al., 2020), in which participants were instructed to insert pegs into a board as quickly as possible using their dominant (9HPT-R) and non-dominant hands (9HPT-L). Mean time (9HPT-M) also was calculated. Severity of depressive symptoms was measured using the Persian-validated version of Beck Depression Inventory-II (BDI-II) (Beck et al., 1996, Ghassemzadeh et al., 2005), a 21-item screening instrument with the following cut-offs; 0–13 (minimal depressive symptoms), 14–19 (mild depressive symptoms), 20–28 (moderate depressive symptoms), and 29–63 (severe depressive symptoms).

### Biochemical Measurements

Venous blood was collected following overnight fasting (≥10 h). Serum samples were immediately centrifuged at 3000 g for 10 min and stored at −70°C until analysis. All assays were performed in duplicate by laboratory personnel blinded to clinical data, using commercially available kits from MyBioSource according to the manufacturer’s instructions. GPx (U/ml) (enzyme-linked immunosorbent assay (ELISA) kit, catalog number: MBS2516156, quantitative sandwich), SOD (U/ml) (ELISA kit, catalog number: MBS2707322, quantitative sandwich), CAT (U/ml) (ELISA kit, catalog number: MBS165657, quantitative sandwich), TAC (mmol/L) (ABTS method, catalog number: MBS2548432), GSH (µmol/L) (ELISA kit, catalog number: MBS265674, sandwich), and MDA (µmol/L) (ELISA kit, catalog number: MBS263626, sandwich) were assessed. The ELISA procedure included overnight incubation of polystyrene plates with diluted capture antibodies, sequential antigen-antibody binding, substrate addition, and absorbance measurement at 450 nm.

### Statistical Analysis

All statistical tests were two-tailed with significance threshold set at p < 0.05. Data analysis was conducted using IBM SPSS Statistics Version 22 (Chicago, IL, USA). Continuous variables were tested for normality using the Kolmogorov–Smirnov test. Normally distributed data are presented as mean ± standard deviation (SD) and non-normally distributed data as median and interquartile range [Q1, Q3]. Categorical variables are presented as frequencies and percentages. Differences between groups were assessed using the independent sample t test, Mann-Whitney *U* test, or chi-square, when appropriate. Potential confounders were adjusted using the multivariate binary logistic regression analysis. Univariate correlations between variables were examined using Spearman rank correlation coefficients, given non-normal distributions of most variables. Heatmap for the correlation matrix between the clinical and laboratory findings of the study was generated using the ninth version of GraphPad Prism. Multivariable linear regression models were constructed to identify independent predictors of investigated outcomes. The hierarchical approach involved: (1) Model 1—entry of demographic variables (age, sex, education); (2) Model 2—addition of clinical (disease duration, EDSS, BDI-FS) and laboratory oxidative stress markers (MDA, TAC, SOD, CAT, GPx, GSH).

## Results

### Characteristics of the participants

The studied cohort comprised 83 RRMS patients with female predominance (77.1%) and mean age of 32.65 ± 8.05 years (range: 18–58). Median disease duration was 60 months (Q1-Q3: 24–96 months, range: 1–240 months), median EDSS was 1.0 (Q1-Q3: 0–2, range: 0–3) and median for education years was 14 years (Q1-Q3: 12–16 years). Employment status was diverse: 55.4% actively employed, 26.5% unemployed, 18.1% retired or engaged in housekeeping. Table 1 presents complete characteristics of the participants.

**Table 1:** – Characteristics of the RRMS patients included in the study.

**Characteristics**	**Overall (n = 83)**	**Min, max**
**Age (years old; mean±SD)**	32.65 ± 8.05	18, 58
**Female sex (number (percentage%))**	64 (77.1%)	-
**Employment status (number (percentage%))**		
**Active** **Retired/housekeeping** **Student**	22 (26.5%) 46 (55.4%) 15 (18.1%)	
**Education (years; median [Q1, Q3])**	14 [12, 16]	9, 18
**Disease duration (months; median [Q1, Q3])**	60 [24, 96]	1, 240
**Disease-modifying therapies (number (percentage%))**		
**DMF** **Fingolimod** **Glatiramer acetate** **Interferon beta 1-a** **Natalizumab** **No drug for MS** **Ocrelizumab** **Rituximab** **Teriflunomide**	32 (38.6%) 15 (18.1%) 1 (1.2%) 11 (13.3%) 3 (3.6%) 4 (4.8%) 6 (7.2%) 9 (10.8%) 2 (2.4%)	
**EDSS (Score; median [Q1-Q3])**	1 [0, 2]	0, 3
**BDI-II (Score; median [Q1-Q3])**	13 [7, 21]	0, 46
**Depressive symptoms (number (percentage%))**		
**Minimal (scores: 0–13)** **Mild (scores: 14–19)** **Moderate (scores: 20–28)** **Severe (scores: 29–63)**	46 (55.4%) 14 (16.9%) 12 (14.5%) 11 (13.3%)	
**9HPT-R (Seconds; median [Q1, Q3])**	23.09 [21.08, 24.67]	16.71, 44.59
**9HPT-L (Seconds; median [Q1, Q3])**	24.05 [22.16, 27.50]	16.68, 53.68
**9HPT-M (Seconds; median [Q1, Q3])**	23.66 [21.62, 26.30]	17.30, 41.55
**SDMT (Score; mean±SD)**	49.64 ± 14.97	20, 110
**Abnormal cognitive function (SDMT≤44; number (percentage%))**	30 (36.1%)	-
**PASAT (score; mean±SD)**	46.62 ± 9.88	22, 60
**GPx (U/ml; median [Q1, Q3])**	226.30 [185.30, 263.50]	98.50, 524.90
**TAC (mmol/L; median [Q1, Q3])**	378.10 [321.50, 461.50]	208.60, 761.00
**CAT (U/ml; median [Q1, Q3])**	36.40 [33.10, 38.40]	19.70, 54.10
**SOD (U/ml; median [Q1, Q3])**	169.50 [128.50, 198.62]	56.80, 311.80
**MDA (µmol/L; median [Q1, Q3])**	2.67 [2.10, 3.13]	0.48, 4.25
**GSH (µmol/L; median [Q1, Q3])**	2.69 [2.34, 3.69]	1.28, 5.81

SD: Standard Deviation; IQR: Interquartile Range; EDSS: Expanded Disability Status Scale; BDI-II: Beck Depression Inventory-II; SDMT: Symbol Digit Modalities Test; PASAT: Paced Auditory Serial Addition Test; GPx: Glutathione peroxidase; TAC: Total Antioxidant Capacity; CAT: Catalase; SOD: Superoxide Dismutase; MDA: Malondialdehyde; GSH: Glutathione.

### Factors associated with depressive symptoms

**Fig. 1:**
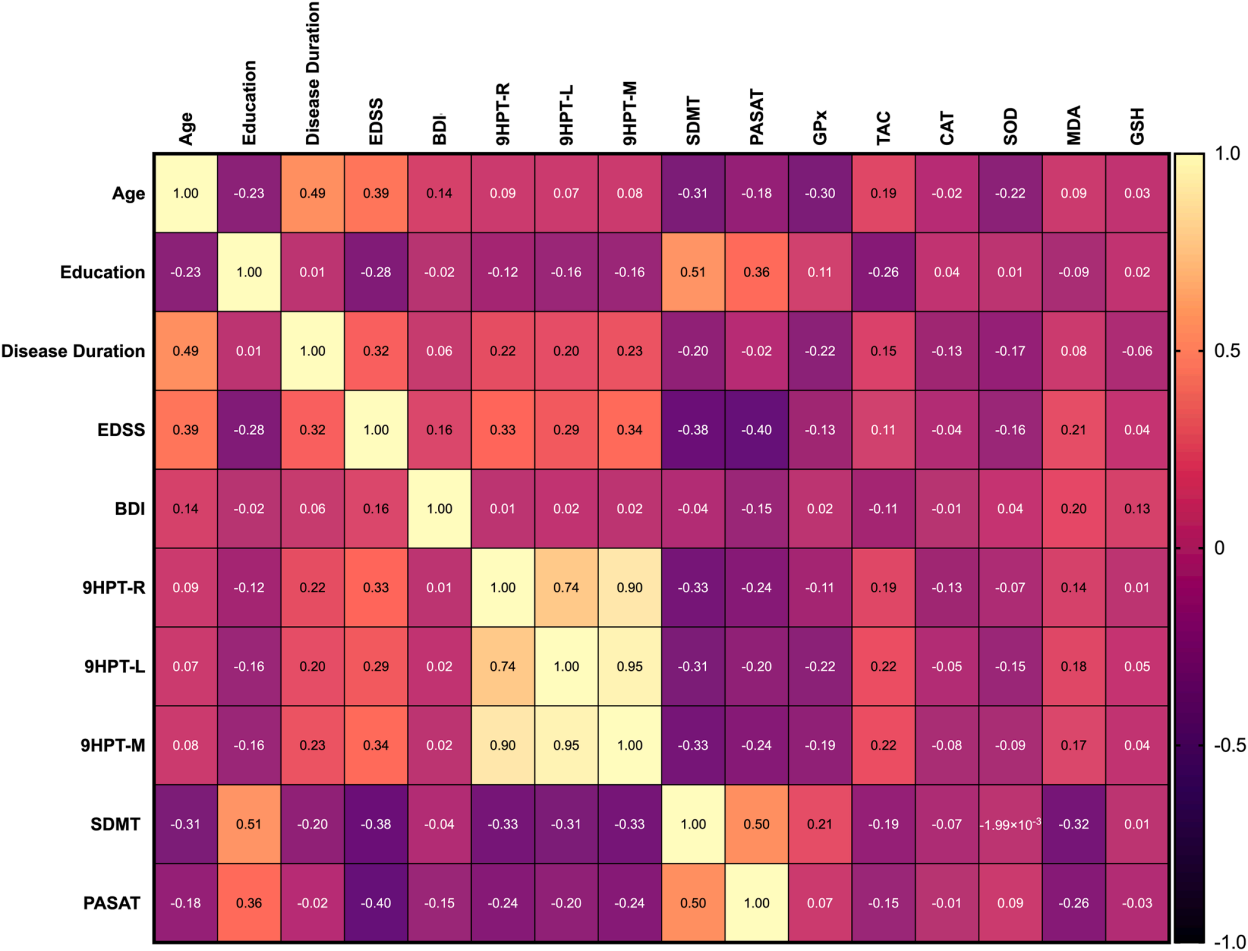
– Correlation matrix between the clinical and laboratory findings of the study (the reported values are correlation ‘r’ based on the Spearman correlation coefficients). EDSS: Expanded Disability Status Scale; BDI-II: Beck Depression Inventory-II; SDMT: Symbol Digit Modalities Test; PASAT: Paced Auditory Serial Addition Test; GPx: Glutathione peroxidase; TAC: Total Antioxidant Capacity; CAT: Catalase; SOD: Superoxide Dismutase; MDA: Malondialdehyde; GSH: Glutathione.

As presented in Table 2, patients with minimal depressive symptoms (n = 46) did not differ significantly from patients with mild/moderate/severe depressive symptoms (n = 37) in none of the investigated factors (p-values>0.05). Multivariate binary logistic regression analysis did not materially alter the results (p-values>0.05). Also, severity of depressive symptoms, based on BDI-II scores showed no significant univariate correlation with other variables (Fig. 1). Multivariable linear regression analysis with BDI-II as the dependent variable also was not associated with significant finding (p-values>0.05).

**Table 2:** – The statistics of the RRMS patients participated in the study in groups based on the depressive symptoms.

Characteristics	Minimal depressive symptoms (n = 46)	Mild/moderate/severe depressive symptoms (n = 37)	P-value
**Age (years old; mean±SD)**	31.98 ± 8.66	33.49 ± 7.24	0.40
**Female sex (number (percentage%))**	34 (73.9%)	30 (81.1%)	0.60
**Employment status (number (percentage%))**			0.49
**Active** **Retired/housekeeping** **Student**	13 (28.3%) 23 (50.0%) 10 (21.7%)	9 (24.3%) 23 (62.2%) 5 (13.5%)	
**Education (years; median [Q1, Q3])**	14 [12, 16]	12 [12, 16]	0.42
**Disease duration (months; median [Q1, Q3])**	59 [24, 96]	60 [24, 96]	0.98
**Disease-modifying therapies (number (percentage%))**			0.64
**DMF** **Fingolimod** **Glatiramer acetate** **Interferon beta 1-a** **Natalizumab** **No drug for MS** **Ocrelizumab** **Rituximab** **Teriflunomide**	19 (41.3%) 9 (19.6%) 0 (0.0%) 5 (10.9%) 2 (4.3%) 1 (2.2%) 4 (8.7%) 4 (8.7%) 2 (4.3%)	13 (35.1%) 6 (16.2%) 1 (2.7%) 6 (16.2%) 1 (2.7%) 3 (8.1%) 2 (5.4%) 5 (13.5%) 0 (0.0%)	
**EDSS (Score; median [Q1-Q3])**	1 [0, 1.25]	1 [0, 2]	0.33
**9HPT-R (Seconds; median [Q1, Q3])**	23.25 [21.21, 24.65]	22.78 [20.73, 25.09]	0.62
**9HPT-L (Seconds; median [Q1, Q3])**	24.70 [22.90, 27.16]	23.71 [21.21, 27.87]	0.48
**9HPT-M (Seconds; median [Q1, Q3])**	23.85 [21.73, 26.37]	23.53 [21.15, 26.66]	0.53
**SDMT (Score; mean±SD)**	51.70 ± 16.49	47.03 ± 12.56	0.12
**Abnormal cognitive function (SDMT≤44; number (percentage%))**	16 (34.8%)	14 (37.8%)	0.82
**PASAT (score; mean±SD)**	48.28 ± 9.63	44.53 ± 9.93	0.09
**GPx (U/ml; median [Q1, Q3])**	226.5 [185.67, 263.50]	223.50 [167.15, 265.05]	0.89
**TAC (mmol/L; median [Q1, Q3])**	380.90 [353.12, 461.82]	365.80 [303.15, 458.35]	0.18
**CAT (U/ml; median [Q1, Q3])**	33.85 [27.42, 36.47]	30.70 [27.80, 36.25]	0.35
**SOD (U/ml; median [Q1, Q3])**	126.50 [106.80, 157.50]	153.20 [99.65, 189.60]	0.22
**MDA (µmol/L; median [Q1, Q3])**	1.96 [1.42, 2.69]	2.35 [1.65, 2.67]	0.34
**GSH (µmol/L; median [Q1, Q3])**	2.08 [1.58, 2.67]	2.37 [1.94, 3.17]	0.11

SD: Standard Deviation; IQR: Interquartile Range; EDSS: Expanded Disability Status Scale; SDMT: Symbol Digit Modalities Test; PASAT: Paced Auditory Serial Addition Test; GPx: Glutathione peroxidase; TAC: Total Antioxidant Capacity; CAT: Catalase; SOD: Superoxide Dismutase; MDA: Malondialdehyde; GSH: Glutathione.

### Factors associated with disability

Patients with EDSS= 0 (n = 32, 38.6%) were classified as having no detectable disability and those with 0 <EDSS≤ 3 (n = 51, 61.4%) were grouped as having measurable disability. When stratified by disability status, patients with 0 <EDSS≤ 3 were significantly older (34.41 ± 8.32 vs. 29.84 ± 6.83 years, p = 0.01), had longer disease duration (median 66 months vs. 58 months, p = 0.02), and had significantly lower education levels (median 16 vs. 12 years, p = 0.01). The difference in SDMT performance was also substantial (43.24 ± 12.63 vs. 56.28 ± 16.88, p < 0.001), as was the difference in PASAT performance (43.70 ± 9.30 vs. 50.97 ± 9.22, p < 0.001) (Table 3). To further examine the structure of associations, binary logistic regression was employed to identify predictors of any measurable disability (0 <EDSS≤3) versus no disability (EDSS=0). The first model, including only demographic variables (age, education, disease duration), showed that education was a protective factor (OR=0.83, 95%CI: 0.70–0.98, p = 0.03). Addition of cognitive variables suggested independent association between EDSS and SDMT (OR=0.95, 95%CI: 0.92–0.99, p = 0.02) and PASAT (OR=.92, 95%CI: 0.89–0.89, p = 0.01). MDA showed a marginal association with EDSS (OR=1.89, 95%CI: 0.97–3.68, p = 0.06), but this association did not reach conventional statistical significance (Table 4). Multivariate binary logistic regression analysis of other oxidative stress markers also did not affect the significance of the findings (data not shown). As presented in Fig. 1, the strong associations between EDSS and both cognitive measures (SDMT: r = −0.38, p < 0.001; and PASAT: r = −0.40, p < 0.001), age (r = 0.39, p < 0.001), education (r = −0.28, p = 0.009), disease duration (r = 0.31, p = 0.004), 9HPT outcomes (p < 0.05) were evident. MDA showed a weak positive association with EDSS (r = 0.21, p = 0.054), that did not reach statistical significance. In multivariable linear regression analysis with EDSS as the dependent variable, when inserting age, sex, education, disease duration, and BDI-II as confounding variables, MDA was the only associated laboratory variable with EDSS (B = 0.30 (95%CI: 0.01, 0.60), SE: 0.15, Standardized Coefficients β: 0.21, p-value:0.04).

**Table 3 –:** The statistics of the RRMS patients included in the study in groups based on the level of disability.

**Characteristics**	**EDSS= 0 (n = 32)**	**0 <EDSS≤ 3 (n = 51)**	**P-value**
**Age (years old; mean±SD)**	29.84 ± 6.83	34.41 ± 8.32	0.01*
**Female sex (number (percentage%))**	24 (75.0%)	40 (78.4%)	0.79
**Employment status (number (percentage%))**			0.07
**Active** **Retired/housekeeping** **Student**	10 (31.3%) 13 (40.6%) 9 (28.1%)	12 (23.5%) 33 (64.7%) 6 (11.8%)	
**Education (years; median [Q1, Q3])**	16 [12, 16]	12 [11.75, 16]	0.01*
**Disease duration (months; median [Q1, Q3])**	58 [12, 72]	66 [24, 120]	0.02*
**Disease-modifying therapies (number (percentage%))**			0.29
**DMF** **Fingolimod** **Glatiramer acetate** **Interferon beta 1-a** **Natalizumab** **No drug for MS** **Ocrelizumab** **Rituximab** **Teriflunomide**	12 (37.5%) 4 (12.5%) 1 (3.1%) 7 (21.9%) 2 (6.3%) 2 (6.3%) 2 (6.3%) 1 (3.1%) 1 (3.1%)	20 (39.2%) 11 (21.6%) 0 (0.0%) 4 (4.7%) 1 (2.0%) 2 (3.9%) 4 (4.7%) 8 (15.7%) 1 (2.0%)	
**BDI-II (Score; median [Q1-Q3])**	11 [7, 19]	13 [7.75, 21.5]	0.35
**Depressive symptoms (number (percentage%))**			0.72
**Minimal** **Mild** **Moderate** **Severe**	19 (59.4%) 5 (15.6%) 3 (9.4%) 5 (15.6%)	27 (52.9%) 9 (17.6%) 9 (17.6%) 6 (11.8%)	
**9HPT-R (Seconds; median [Q1, Q3])**	21.62 [20.73, 24.40]	23.49 [21.28, 26.14]	0.07
**9HPT-L (Seconds; median [Q1, Q3])**	23.42 [21.56, 26.58]	25.08 [22.38, 27.64]	0.17
**9HPT-M (Seconds; median [Q1, Q3])**	23.04 [21.10, 24.66]	23.99 [22.12, 26.86]	0.07
**SDMT (Score; mean±SD)**	56.28 ± 16.88	43.24 ± 12.63	< 0.01*
**Abnormal cognitive function (SDMT≤44; number (percentage%))**	6 (18.8%)	24 (47.1%)	0.01*
**PASAT (score; mean±SD)**	50.97 ± 9.22	43.70 ± 9.30	< 0.01*
**GPx (U/ml; median [Q1, Q3])**	226.70 [159.80, 262.77]	222.5 [185.60, 263.50]	0.88
**TAC (mmol/L; median [Q1, Q3])**	366.00 [308.62, 480.52]	385.40 [341.00, 451.80]	0.61
**CAT (U/ml; median [Q1, Q3])**	32.60 [28.30, 34.80]	34.70 [27.30, 36.50]	0.62
**SOD (U/ml; median [Q1, Q3])**	129.45 [114.57, 168.52]	127.80 [96.50, 185.20]	0.59
**MDA (µmol/L; median [Q1, Q3])**	1.66 [1.40, 2.64]	2.35 [1.65, 2.68]	0.10
**GSH (µmol/L; median [Q1, Q3])**	2.35 [1.67, 2.66]	2.31 [1.67, 2.69]	0.92

SD: Standard Deviation; IQR: Interquartile Range; EDSS: Expanded Disability Status Scale; BDI-II: Beck Depression Inventory-II; SDMT: Symbol Digit Modalities Test; PASAT: Paced Auditory Serial Addition Test; GPx: Glutathione peroxidase; TAC: Total Antioxidant Capacity; CAT: Catalase; SOD: Superoxide Dismutase; MDA: Malondialdehyde; GSH: Glutathione.

**Table 4:** – Multivariate binary logistic regression analysis of the predictors of disability (0 <EDSS≤3) in RRMS patients participated in the study.

**Characteristics**	**Odds ratio (95% confidence intervals)**	**P-value**
***First model***		
**Age**	1.04 (0.97, 1.11)	0.30
**Education**	0.83 (0.70, 0.98)	0.03*
**Disease duration**	1.01 (0.99, 1.03)	0.05
***Second model***		
**Age**	1.03 (0.96, 1.12)	0.40
**Education**	0.88 (0.73, 1.06)	0.19
**Disease duration**	1.01 (0.99, 1.02)	0.13
**SDMT**	0.95 (0.92, 0.99)	0.02*
***Third model***		
**Age**	1.02 (0.94, 1.10)	0.62
**Education**	0.89 (0.74, 1.09)	0.26
**Disease duration**	1.01 (1.00, 1.03)	0.03*
**PASAT**	0.92 (0.86, 0.89)	0.01*
**Fourth model**		
**Age**	1.03 (0.96, 1.11)	0.39
**Education**	0.83 (0.70, 0.98)	0.02*
**Disease duration**	1.01 (1.00, 1.02)	0.05
**MDA**	1.89 (0.97, 3.68)	0.06

SDMT: Symbol Digit Modalities Test; PASAT: Paced Auditory Serial Addition Test; MDA: Malondialdehyde.

### Factors associated with visual information processing speed

Patients with SDMT≤ 44 were significantly older (35.8 ± 8.42 vs. 30.87 ± 7.32 years, p < 0.01), less likely to be employed (p < 0.05), less educated (median 12 years vs. median 16 years, p < 0.01), and have extended disease duration (median 72 months vs. 42 months, p < 0.01), and higher EDSS score (p < 0.01). In addition, time to complete 9HPT-R test and 9HPT-M were higher in patients with SDMT≤ 44 (both p = 0.03). MDA also was significantly higher (median 2.65 µmol/L vs. median 1.84 µmol/L, p < 0.01) in SDMT≤ 44 group (Table 5). Multivariate binary logistic regression further identified education as a consistent protective element across models (ORs ranging from 0.68 to 0.73, p < 0.01), disease duration as a modest risk factor (OR: 1.01, p = 0.03–0.04), and MDA as an independent predictor in the final model (OR: 2.98, 95% CI: 1.33–6.66, p < 0.01) (Table 6). Multivariate binary logistic regression analysis of other oxidative stress markers did not affect the significance of the findings (p-values>0.05). As presented in Fig. 1, the correlation between SDMT scores and age (r = −0.31, p = 0.04), education (r = 0.51, p < 0.001), EDSS (r = −0.38, p < 0.001), 9HPT outcomes (p < 0.05), and MDA (r = −0.32, p = 0.003) were evident. In multivariable linear regression analysis with SDMT as the dependent variable, among the included variables, education level (B = 1.35 (95%CI: 0.37, 2.33), SE: 0.49, Standardized Coefficients β: 0.29, p-value< 0.01), and EDSS (B = −3.15 (95%CI: −6.25, −0.04), SE: 1.56, Standardized Coefficients β: −0.23, p-value: 0.047) were the only independent predictors of SDMT scores and age, sex, disease duration, and BDI-II were not independently associated. When controlling for confounding factors, no independent association between laboratory markers of the study and SDMT scores were observed and the univariate correlation between MDA and SDMT was no longer statistically significant after adjustment (B = −4.07 (95%CI: −8.17, 0.02), SE: 2.05, Standardized Coefficients β: −0.21, p-value: 0.051). In addition, no significant association between the SDMT and 9HPT-M scores was observed, when controlling for confounders (B = −0.76 (95%CI: −1.54, 0.01), SE: 0.39, Standardized Coefficients β: −0.23, p-value: 0.053).

**Table 5:** – The statistics of the RRMS patients participated in the study in groups based on cognitive function.

**Characteristics**	**SDMT≤ 44 (n = 30)**	**SDMT> 44 (n = 53)**	**P-value**
**Age (years old; mean±SD)**	35.8 ± 8.42	30.87 ± 7.32	< 0.01*
**Female sex (number (percentage%))**	24 (80.0%)	40 (75.5%)	0.79
**Employment status (number (percentage%))**			0.05*
**Active** **Retired/housekeeping** **Student**	5 (16.7%) 22 (73.3%) 3 (10.0%)	17 (31.1%) 24 (45.3%) 12 (22.6%)	
**Education (years; median [Q1, Q3])**	12 [9, 12.5]	16 [12, 16]	< 0.01*
**Disease duration (months; median [Q1, Q3])**	72 [39, 123]	42 [16.5, 84]	0.02*
**Disease-modifying therapies (number (percentage%))**			0.78
**DMF** **Fingolimod** **Glatiramer acetate** **Interferon beta 1-a** **Natalizumab** **No drug for MS** **Ocrelizumab** **Rituximab** **Teriflunomide**	21 (39.6%) 9 (17.0%) 1 (1.9%) 8 (15.1%) 3 (5.7%) 2 (3.8%) 4 (7.5%) 4 (7.5%) 1 (1.9%)	11 (36.7%) 6 (20.0%) 0 (0.0%) 3 (10.0%) 0 (0.0%) 2 (6.7%) 2 (6.7%) 5 (16.7%) 1 (3.3%)	
**EDSS (Score; median [Q1-Q3])**	1 [1, 2]	1 [0, 1]	< 0.01*
**BDI-II (Score; median [Q1-Q3])**	12 [6, 24]	13 [9, 20]	0.63
**Depressive symptoms (number (percentage%))**			0.56
**Minimal** **Mild** **Moderate** **Severe**	16 (53.3%) 4 (13.3%) 4 (13.3%) 6 (20.0%)	30 (56.6%) 10 (18.9%) 8 (15.1%) 5 (9.4%)	
**9HPT-R (Seconds; median [Q1, Q3])**	23.71 [21.27, 29.02]	22.69 [21.00, 24.04]	0.03*
**9HPT-L (Seconds; median [Q1, Q3])**	26.19 [22.70, 29.05]	23.75 [21.39, 26.36]	0.06
**9HPT-M (Seconds; median [Q1, Q3])**	24.12 [22.07, 28.58]	23.45 [21.38, 24.73]	0.03*
**PASAT (score; mean±SD)**	41.28 ± 8.86	49.19 ± 9.37	< 0.01*
**GPx (U/ml; median [Q1, Q3])**	201.90 [175.70, 256.07]	235.80 [195.60, 264.60]	0.10
**TAC (mmol/L; median [Q1, Q3])**	385.50 [363.35, 466.75]	365.80 [306.50, 457.80]	0.15
**CAT (U/ml; median [Q1, Q3])**	34.80 [28.37, 36.42]	31.90 [26.55, 35.90]	0.25
**SOD (U/ml; median [Q1, Q3])**	126.60 [100.22, 172.67]	129.40 [102.00, 172.50]	0.61
**MDA (µmol/L; median [Q1, Q3])**	2.65 [1.87, 2.86]	1.84 [1.48, 2.54]	< 0.01*
**GSH (µmol/L; median [Q1, Q3])**	2.28 [1.81, 2.67]	2.37 [1.67, 2.73]	0.90

SD: Standard Deviation; IQR: Interquartile Range; EDSS: Expanded Disability Status Scale; BDI-II: Beck Depression Inventory-II; SDMT: Symbol Digit Modalities Test; PASAT: Paced Auditory Serial Addition Test; GPx: Glutathione peroxidase; TAC: Total Antioxidant Capacity; CAT: Catalase; SOD: Superoxide Dismutase; MDA: Malondialdehyde; GSH: Glutathione.

**Table 6 –:** Multivariate binary logistic regression analysis of the predictors of SDMT≤ 44 in RRMS patients participated in the study.

**Characteristics**	**Odds ratio (95% confidence intervals)**	**P-value**
***First model***		
**Age**	1.03 (0.96, 1.11)	0.37
**Education**	0.71 (0.58, 0.86)	< 0.01*
**Disease duration**	1.01 (1.00, 1.02)	0.03*
***Second model***		
**Age**	1.03 (0.96, 1.11)	0.40
**Education**	0.71 (0.58, 0.87)	< 0.01*
**Disease duration**	1.01 (1.00, 1.02)	0.04*
**EDSS**	1.02 (0.63, 1.66)	0.94
***Third model***		
**Age**	1.02 (0.95, 1.10)	0.55
**Education**	0.73 (0.60, 0.88)	< 0.01*
**Disease duration**	1.01 (0.99, 1.02)	0.07
**9HPT-M**	1.11 (0.97, 1.26)	0.14
**Fourth model**		
**Age**	1.03 (0.96, 1.11)	0.40
**Education**	0.68 (0.56, 0.84)	< 0.01*
**Disease duration**	1.01 (1.00, 1.02)	0.03*
**MDA**	2.98 (1.33, 6.66)	< 0.01*

EDSS: Expanded Disability Status Scale; PASAT: Paced Auditory Serial Addition Test; MDA: Malondialdehyde.

### Factors associated with auditory information processing speed

As presented in Fig. 1, PASAT scores were significantly correlated with education (r = 0.36, p = 0.001), EDSS (r = −0.40, p < 0.001), 9HPT-R (r = −0.24, p = 0.03), 9HPT-M (r = −0.24, p = 0.03), and MDA (r = −0.26, p-value=0.02). In multivariable linear regression analysis with PASAT as the dependent variable, among the included variables, sex (B = 8.08 (95%CI: 3.57, 12.59), SE: 2.26, Standardized Coefficients β: 0.36, p-value< 0.01), education level (B = 0.82 (95%CI: 0.19, 1.46), SE: 0.32, Standardized Coefficients β: 0.27, p-value: 0.01), and EDSS (B = −2.94 (95%CI: −4.76, −1.13), SE: 0.91, Standardized Coefficients β: −0.36, p-value: 0.002) were the independent predictors of PASAT scores. Age, disease duration, and BDI-II were not independently associated with PASAT. When controlling for confounding factors, no significant association between the laboratory findings of the study and PASAT scores were observed and the association between MDA and PASAT lost significance (B = −0.93 (95%CI: −3.75, 1.88), SE: 1.41, Standardized Coefficients β: −0.07, p-value: 0.051). In addition, no significant association between the PASAT and 9HPT-M scores were observed, when controlling for confounders (B=−0.30 (95%CI: −0.79, 0.19), SE:0.24, Standardized Coefficients β: −0.14, p-value: 0.225).

### Factors associated with Manual Dexterity

As presented in Fig. 1, 9HPT-M was significantly correlated with EDSS (r = 0.34, p = 0.002), SDMT (r = −0.33, p = 0.002), PASAT (r = −0.24, p = 0.03), and TAC (r = 0.22, p = 0.04). In multivariable linear regression analysis with 9HPT-M as the dependent variable, among the included variables, EDSS (B = 1.76 (95%CI: 0.84, 2.68), SE: 0.46, Standardized Coefficients β: 0.43, p-value< 0.001) was the only independent predictor of 9HPT-M. Age, sex, education, disease duration, and BDI-II were not independently associated with 9HPT-M. When controlling for confounding factors, TAC remained independently associated with 9HPT-M (B = 0.01 (95%CI: 0.003, 0.021), SE: 0.005, Standardized Coefficients β: 0.26, p-value: 0.01). No other significant association between the laboratory as well as cognitive findings of the study and 9HPT-M were observed (p-values>0.05).

## Discussion

This study investigated the relationships between the clinical and biochemical outcomes in a cohort of RRMS patients with mild/moderate disability. Based on the findings of this study, both visual and auditory information process speed were independently associated disability status, consistent with the strong bidirectional association between cognitive function and disability (Gois et al., 2021), even after adjustment for possible confounders. Education emerged as a strong protective factors for cognitive dysfunction, consistent with cognitive reserve theory, the capacity to maintain cognitive performance through compensatory neural mechanisms (Zhu et al., 2021). No clinically significant correlations were observed between any oxidative stress marker and neuropsychological outcomes, including the depressive symptoms and information process speed as well as level of disability, according to EDSS. Antioxidant supplementations such as melatonin (Morsali et al., 2023), omega−3 fatty acids (AlAmmar et al., 2021), probiotics (Eghbal and Naseri, 2026, Mosaddeghi-Heris et al.,), and coenzyme Q10 (Salekzamani et al., 2025), have attracted increasing interest as a complementary strategy in MS; However, the absence of independent associations between peripheral oxidative stress markers and cognitive or disability outcomes in the present study suggests that circulating oxidative stress biomarkers should not be considered reliable surrogate markers for selecting patients or monitoring the response to antioxidant-based interventions, particularly in early-stage of RRMS.

The predominance of female participants in our cohort is consistent with the well-established epidemiology of MS, which affects women more frequently than men (D’Anca et al., 2023). In case of MS, male sex is suggested to be associated with faster disability progression and a more aggressive disease course (Ahmadi Bonabi et al., 2026). In addition, association between sex and MS symptoms such as sleep problems (Yazdchi et al., 2022), cognitive outcomes (Naseri et al., 2022, Talebi et al., 2022), fatigue, and depressive symptoms (Gottwald et al., 2024), are inconsistently reported. In this study, male sex was independently associated with better working memory and auditory information processing speed (PASAT scores) after adjustment for potential confounders, which warrants further evaluations.

The biological mechanisms underlying MS-related cognitive impairment remain incompletely understood, and accumulating evidence suggests potential roles for neuroinflammation (Rademacher et al., 2023), neurodegeneration, particularly in progressive forms of the disease (Benedict et al., 2020), disrupted functional brain networks (Rahnemayan et al., 2025), oxidative stress and inflammation (Daneshvar et al., 2025), alterations in cholesterol metabolism (Sanaie et al., 2024, Siddiqui et al., 2023) and genetic susceptibility (Mashayekhi et al., 2022, Naseri et al., 2022). Regarding the oxidative stress, in a previous research of the current team in 71 MS patients, including both RRMS and progressive forms of the disease and wider range of EDSS (between 0 and 7.5), no significant association was detected between oxidative stress markers (including CAT, GPX1, GPX2, GSH, TAC, SOD, and MDA) and cognitive outcomes, based on Minimal Assessment of Cognitive Function in MS (MACFIMS) battery (Naseri, Forghani, et al., 2022). In another study involving 65 patients with all subtypes of MS, correlations between the TAC as well as MDA and Cambridge neuropsychological test automated battery (CANTAB) outcomes were observed (Talebi et al., 2021). Regarding other markers of oxidative stress, in Can Demirdöğen et al.’s study of 85 patients with different types of MS, thiol-disulfide homeostasis and ischemia-modified albumin as two biochemical biomarkers of oxidative stress, were found to be associated with Brief International Cognitive Assessment for MS (BICAMS) test outcomes. SDMT scores in this study were correlated with ischemia-modified albumin/albumin ratio (Can Demirdöğen et al., 2023). Correlation between 4-HNE, TAC and processing speed, based on SDMT, were also reported in Piñar-Morales et al.’s study of 77 RRMS patients (Piñar-Morales et al., 2025). In this research, no clinically significant correlations were observed in this regard. Considering the lack of independent associations between the investigated oxidative stress markers and cognitive outcomes, together with the strong association between cognitive performance and EDSS, our findings suggest that cognitive impairment in RRMS is more closely related to neurological disability than to peripheral oxidative stress, even among fully ambulatory patients with mild/moderate disability. Longitudinal studies incorporating repeated cognitive assessments and serial biomarker measurements are warranted to clarify the temporal relationships.

This study controlled for EDSS in regression models, yet depression-cognition associations remained absent, supporting a lack of association rather than residual confounding. Considering the well-defined association between the depression and cognitive function (Kriesche et al., 2023, Zacková et al., 2021), the absence of significant associations in this study can be attributed to the relatively low burden of depressive symptoms in our cohort.

The relationship between hand dexterity and cognitive function in MS has been minimally investigated. Regarding 9HPT, patients with SDMT≤ 38 differed significantly with SDMT> 38 in Piñar-Morales et al.’s study of 77 RRMS patients (Piñar-Morales et al., 2025). Similarly, in our study a significant association was observed; however, it was no longer evident when after adjustment for potential confounders. In another evaluation of 60 MS patients, hand dexterity, evaluated by the Purdue Pegboard Test, was identified as a predictor of disability status, based on EDSS (Ghandi Dezfuli et al., 2015), a finding consistent with our results.

This study employed comprehensive neuropsychological assessment, validated clinical disability measures, carefully standardized biochemical assays, and multivariable statistical approaches accounting for multiple confounders. Although no formal a priori sample size calculation was performed, the study included all eligible consecutive patients during the recruitment period. The cross-sectional design precludes causal inference and temporal ordering of the observed associations. Longitudinal multi-center studies with larger sample sizes and with longitudinal assessment of cognitive trajectories and biomarker evolution over years are warranted. Considering the early-stage disease and RRMS phenotype, the findings of this research cannot be generalized to patients with major disability and/or progressive forms of the disease.

## Conclusion

This cross-sectional study of RRMS patients with mild/moderate disability demonstrates that physical disability and education level are important predictors of cognitive performance, while peripheral oxidative stress markers, do not independently predict information processing speed. Future longitudinal and neuroimaging studies are warranted to clarify the temporal relationships between central and peripheral oxidative stress, neurodegeneration, and cognitive decline in MS.

## Ethics Approval

The study protocol was approved by the Ethics Committee of Tabriz University of Medical Sciences (Ethics code: IR.TBZMED.REC.1404.897).

## Funding

This research was funded by the Student Research Committee of 10.13039/501100004366Tabriz University of Medical Sciences (Grant No: 76354).

## CRediT authorship contribution statement

**Niloofar Taheri:** Writing – review & editing, Visualization, Methodology, Formal analysis, Data curation. **Mahnaz Talebi:** Writing – review & editing, Validation, Supervision, Resources, Project administration, Methodology, Investigation, Funding acquisition, Conceptualization. **Reza Mosaddeghi-Heris:** Writing – original draft, Visualization, Investigation, Data curation. **Amirreza Naseri:** Writing – review & editing, Visualization, Resources, Project administration, Methodology, Investigation, Funding acquisition, Formal analysis, Data curation, Conceptualization.

## Consent for Publication and Participation

Written consent was obtained from all participants prior to study inclusion, including consent for the publication of anonymized data.

## Declaration of Competing Interest

The authors declare that they have no known competing financial interests or personal relationships that could have appeared to influence the work reported in this paper.
